# The Therapeutics of Oxygen Gas

**Published:** 1892-03-19

**Authors:** 


### THE PRACTITIONER'S MIRROR AND RETROSPECT.

[*The Editor will be glad to receive offers of co-operation and contributions from members of the profession. All letters should be addressed to* THE EDITOR, THE LODGE, PORCHESTER SQUARE, LONDON, W.]

### MEDICINE.

### THE THERAPEUTICS OF OXYGEN GAS.

The recent appearance of several reports of cases treated by the inhalation of oxygen gas revives once more hopes of the "great things" from its use that were entertained at the end of the last century.

It is not surprising that the properties of oxygen gas should have suggested its use in disease, but past experience has been disappointing, and it is well to bear in mind that it is by no means the harmless remedy that it appears to be thought. We note that in 1864 MM. Demarquay and Leconte* communicated to the Academy of Science, Paris, the results of their researches with respect to oxygen. From their observations on animals they found that dogs were able to respire, during a long period, thirty or forty litres of oxygen and more, the only effect of the inhalations being to render the animal more lively, and to increase his appetite. Wounds which were undergoing the process of repair were observed to become brightly injected during the inhalation of the oxygen, and a serous fluid exuded from the surface, while, as the amount of oxygen inhaled increased, petechial spots appeared. They came to the following conclusions: 1. That rabbits have lived from fourteen to seventeen hours in an atmosphere of oxygen. 2. After death the muscular system of these animals was found extremely turgescent. 3. Contrary to the opinion of Broughton, the various arterial systems retained their natural colour. 4. Contrary to the assertion of Beddoes, no organ, however vascular it be, was ever found the seat of inflammation or gangrene. 5. The muscular system assumed an entirely peculiar rose colour.

These observers, their pupils and friends, then proceeded to make observations on themselves. They found that they could inspire a dose of from twenty to thirty litres without inconvenience. Daily inhalations of from twenty to forty litres during a month or six weeks gave rise to a slight warmth of the fauces and chest, sometimes accompanied by a little intoxication and headache. In most persons the pulse at first increases in number, but in some it becomes slower; and in general the inhalation is followed by an increase of strength and appetite. But patients exhausted by prolonged disease did not show these modifications, and in those who had wounds, recent or old, a few days' inhalation rendered them red and tinged, and more freely suppurating. It was remarked that this peculiar action on wounds explains the results obtained in phthisis by Chaptal and Fourcoy. Patients in the third stage of the disease at first derived great benefit from the inhalations, but the inflammatory symptoms soon becoming more intense, with abundant expectoration and more frequent cough, death soon following. The following are laid down as contra-indications for the use of oxygen inhalations: the febrile condition, except under certain diathetic conditions, as croup; deep-seated, inflammatory action and visceral lesions; diseases of the heart and large vessels, neuralgia unconnected with anæmia, and a disposition to hæmorrhage. On the other hand they believed that oxygen is especially indicated for anæmia or chloro-anæmia connected with surgical affections, for raising the strength, and for combating certain diseases, the depressing action of which is well known, as diphtheritis, syphilis, diabetes, &c. In these conditions, under the influence of oxygen, in the course of a few days, if the age and general condition still allow of it, the strength is recruited, and the appetite returns with intensity.

* *Med. Times and Gaz.*, 1864; Feb. 13th, 27th, Mar. 26th.

The remedial use of oxygen inhalations, has, till quite recently, been almost discontinued. This, no doubt, is partly due to the difficulty that has hitherto existed in obtaining a supply of oxygen gas at reasonable rates, a difficulty which no longer holds. As a quick and effectual cardiac stimulant it merits more attention than we have been bestowing on it, and it will prove useful in cases of asphyxia from carbonic acid poisoning, in chloroform narcosis, and other conditions. "In the suffocation of angina pectoris, the beneficial action of the gas has been abundantly proved, and among other cases occurring latterly was that of General Sheridan, in which the treatment followed was like that of Dr. Robert Reid, of Dublin, who, in 1817, used and advocated the gas as a practical remedy for this affection. One of Dr. Reid's cases was that of a man, aged sixty-four, who had suffered greatly from angina, and who obtained an amount of relief from oxygen inhalation that no other agency gave him. The effect of the gas is, however, generally temporary, but life may often be saved by a temporary tiding over of the suffocative attack. The writer quotes from a recent number of the *Nineteenth Century* a case of gas poisoning which was successfully treated by oxygen inhalation. A soldier was found apparently lifeless and pulseless in consequence of having been exposed for a considerable time to coal gas from a burst balloon. An officer of his command bethought himself of a bottle of compressed oxygen as a possible antidote. A tube having been attached to the bottle, its mouthpiece was conveyed to the man's mouth. The oxygen was liberated through the tube and appeared to force its way into the man's lungs and to become an immediate stimulant to the respiratory organs. In from ten to fifteen seconds from the first outrush of the oxygen gas the man, who had just before presented the aspects of a livid corpse, became agitated with paroxysms of a violence so marked that it was deemed expedient to order four of his comrades to hold him quiet. A half hour later the man was calmly walking back to the barracks, all danger being at an end. The writer accepts both the authenticity and promptness of the recovery in this case as a lesson in the treatment of cases of poisoning with carbon gases, especially those of the methane series. He also commends it to the attention of all who are concerned in the administration of anæsthetics, and remarks: 'How much better a whiff of pure oxygen than artificial respiration in chloral poisoning and chloroform narcosis!'" *

Our remarks and abstracts from the older literature of oxygen inhalations will not be without interest in connection with more recent communications on the subject. Dr. Lauder Brunton gives a concise review of the therapeutics of oxygen in his text-book, but the report of the experience of Drs. Brunton and Prickett of oxygen and strychnine in pneumonia give fresh interest to the subject. They say it is self-evident that if we can increase the oxygenating power of the air inhaled by the patients in cases where the breathing surface of the lung is diminished, we may afford great benefit, and in some cases may save life. "More especially is this likely to be useful when the interference with respiration is of a temporary character, as in cases of acute pneumonia. In some cases where one lung, or one part of a lung, is closing up, while another part is becoming involved, the question of life or death will be decided by the amount of lung available for

* *New York Med. Jour.*, Oct. 24th, 1891; *Med. Press and Circ.*, June 3rd, 1891.

respiration. This, again, will depend upon the comparative rate with which the inflammation encroaches on the breathing space on the one hand, and the already consolidated lung on the other." Dr. Lauder Brunton recently advocated strychnine in snake poisoning, and strychnine has been lauded as a cardiac stimulant. His combination of strychnine with oxygen inhalations seems to increase the likelihood of obtaining a successful result in certain desperate cases. We fear, however, that Dr. Brunton's advocacy of strychnine has tended to make it a fashionable panacea in the hands of those whose therapeutical resources and discrimination are less than his. Dr. Brunton's case was that of a clergyman who was attacked with influenza and pneumonia of the right base, and it was considered impossible for him to live more than two hours. Two hypodermic injections of strychnine, each of one-fortieth of a grain, were given in order to stimulate the respiratory centre, but without much result. Dr. Brunton had previously given oxygen inhalations in respiratory failure in snake bite, and he hoped that its action would preserve life in the present case. About two hours after the inhalations were begun an extraordinary transformation had taken place. The patient, from being unconscious and moribund, was quite conscious, his colour from being livid became normal, and he expressed himself as feeling comfortable and well. The improvement continued after the inhalations were discontinued for about twelve hours. The respiration again failed and twenty-four hours after he had seen him first he was as bad as ever, and in spite of inhalations never recovered again Dr. Brunton and Dr. Prickett remark, "It is possible that nothing could have saved him, but we regretted that we were not summoned when the symptoms became worse as we might have possibly done good by artificial respiration with oxygen." Mr. Langton* has tried oxygen inhalations in severe bronchitis, and though some promising improvement was observed as a result, the patient recovering consciousness, death soon ensued. Mr. Langton attributes the unsuccessful termination in the case of his patient to the accumulation of bronchial secretion in the tubes. He thinks that if she had had sufficient strength to vomit after the emetic he had given, and so have cleared the bronchial tubes, she might have recovered. Dr. Skerritt also reports a case of bronchitis and emphysema in whom temporary improvement was observed following the inhalations. He refers to the case of pneumonia reported by Dr. Blodgett in the *Boston Medical and Surgical Journal*, in which the influence of oxygen is said to have been "almost as pronounced and evident as in that of ligature in hæmorrhage." Mr. Blakiston† has had a more happy experience with this agent, and has been using it for some time past. He administered oxygen in three cases of pneumonia, and one of acute bronchitis; all were unusually severe cases, and all recovered, and he firmly believes that life was saved in two of those cases by the use of oxygen. He has also used oxygen in three cases of asthma. The result in two of the cases was so satisfactory that he is led to strongly recommend an extended trial of the treatment. He has had some experience of oxygen in convalescence from long illness, combined with massage. His more extended and varied experience of oxygen give value to his conclusions: "In oxygen I believe that we have a remedy of great therapeutic value, not solely confined to the treatment of diseases of the respiratory organs. If we only consider for a moment its importance to us in its natural state in the air, and think how many of our patients, especially those in London and other large towns, fail to get their due proportion of it, it is only natural that it should be so. In massage and electricity, combined with the administration of oxygen, we have one of the best substitutes to the convalescent and debilitated constitution for change, exercise, and sea air."

* *Ibid*, January 30, 1892. † *Ibid*, February 6, 1892.

